# Vision Transformer with hierarchical structure and windows shifting for person re-identification

**DOI:** 10.1371/journal.pone.0287979

**Published:** 2023-06-30

**Authors:** Yinghua Zhang, Wei Hou

**Affiliations:** 1 College of Science and Engineering, Jiaozuo Normal College, Jiaozuo, Henan, China; 2 College of Artificial Intelligence, Henan University, Zhengzhou, Henan, China; Anhui University, CANADA

## Abstract

Extracting rich feature representations is a key challenge in person re-identification (Re-ID) tasks. However, traditional Convolutional Neural Networks (CNN) based methods could ignore a part of information when processing local regions of person images, which leads to incomplete feature extraction. To this end, this paper proposes a person Re-ID method based on vision Transformer with hierarchical structure and window shifting. When extracting person image features, the hierarchical Transformer model is constructed by introducing the hierarchical construction method commonly used in CNN. Then, considering the importance of local information of person images for complete feature extraction, the self-attention calculation is performed by shifting within the window region. Finally, experiments on three standard datasets demonstrate the effectiveness and superiority of the proposed method.

## Introduction

Person re-identification (Re-ID) aims to find the target person in a series of images generated by multiple non-overlapping cameras covering a wide area [1]. As an important component of security surveillance and criminal investigations, the person Re-ID has attracted wide attention from researchers in related fields. The biggest challenge of person Re-ID lies in extracting rich, discriminative and robust features from person images, yet this challenge is exacerbated by the presence of many variations in person images such as occlusion, illumination, pose and background clutter.

In recent years, with the development of deep learning technology, computer vision tasks such as image classification, image segmentation, and target tracking have used Convolutional Neural Networks (CNN) as the backbone network for feature extraction, which has promoted researchers to explore more effective CNN-based methods applied to person Re-ID tasks. Among the many CNN-based methods, residual network is more commonly used. The residual network integrates multi-level features by means of jump-connected aggregation, and can better alleviate the gradient disappearance problem. However, due to the Gaussian distribution of the effective receptive field [2], CNN-based methods focus on a small discriminative region and cannot extract richer person image features. To solve the above problems, researchers begun to explore the attention mechanism that relies on large-scale receptive fields to extract information and apply it to person Re-ID. The purpose of the attention mechanism is to find the regions that have a greater impact on the feature map and enhance the model’s focus on local regions [3]. Although some results have been achieved by adding the attention mechanism to CNNs, these approaches embed attention into the deeper layers of CNNs and only work on images with large sizes and continuous regions, without fundamentally solving the problems that exist in CNNs, and it is difficult to extract multiple features with discriminative properties.

Recently, Transformer has been the benchmark model in Natural Language Processing (NLP) with great success. Later, researchers have extended Transformer to computer vision tasks and demonstrate that Transformer can extract effective features and perform various computer vision tasks as well as CNNs. Unlike CNN approaches that focus on extracting hierarchical features, the information interaction in Transformer aims to aggregate features at different scales from the global view. Transformer introduces multi-head attention mechanism and removes the downsampling operator, this design not only can capture a large range of information when extracting image features and drive the model to notice more diverse internal image features than CNN, but also removes the downsampling operator to retain more image information. The single Transformer model lacks some exploitable properties such as shifting and hierarchy to further improve the performance. In addition to the disadvantages mentioned above, when facing person images with high resolution in person Re-ID task, it will bring more computational effort because of its own global self-attention mechanism.

This paper explores how to aggregate multi-level features and save computational effort, so as to carry out person Re-ID tasks more effectively. Also, to address the problems mentioned above, this paper proposes a person Re-ID method based on vision Transformer with hierarchical structure and windows shifting. The attention calculation is restricted to one window, the hierarchical properties of feature representation is considered, and the selection of the backbone network is independent. The main contributions of this paper are summarized as follows.

1. A vision Transformer-based model was proposed for person Re-ID, which aggregates multi-scale person image information while generating discriminative features, achieving better results than CNN-based methods.
2. A method is proposed to extract person image features based on vision Transformer with hierarchical structure and windows shifting, which expands the model’s perceptual field of person images layer by layer and obtains more comprehensive person features while saving computational effort.
3. An extended ablation experiment and complexity analysis experiment is constructed to demonstrate that the proposed method can effectively learn discriminative features. The proposed method is also experimented on three publicly available datasets, and the results achieve excellent performance.

## Related work

The purpose of feature extraction is to obtain discriminative features, which is also a key step in person Re-ID. It is a common practice to design robust deep learning models to learn the overall features of person images from a large amount of training data, and then update the network parameters by optimizing a reasonable loss function to complete feature extraction and similarity measure simultaneously in one framework.

Shao et al. [4] proposed a person Re-ID method that fuses CNN features and attribute features. Although this method complements global and attribute features with each other to accomplish a more comprehensive description of person images, the CNN model has limited ability to extract features and requires a large amount of additional attribute annotation. With the development of deep learning, network models started to shift from general CNNs to more effective models with attention mechanisms.

The role of introducing attention mechanism in CNN is to suppress irrelevant features while enhancing those discriminative features. Song et al. [5] utilized a binary mask attention mechanism to reduce the background noise of person images and enhance the representation of foreground features. Chen et al. [6] proposed a hybrid higher-order attention network which the second-order correlation of features can be obtained to enhance discriminative features. Chen et al. [7] integrated a pair of complementary attention modules to hide features and weights simultaneously by orthogonal normalization and proposed a network called ABD-Net to learn better features. However, the above methods focus only on global features, which is not the optimal case. In person Re-ID, the local information of the image is also discriminative and effective. To solve this problem, the Transformer model, which considers both global and local information, is applied to person Re-ID in this paper.

Recently, Transformer and its variants, which are a fusion attention mechanisms, have received much attention. Transformer is mainly designed based on computer vision tasks such as image classification [8], target detection [9], and image segmentation [10], but it cannot be fully adapted to person Re-ID tasks. Therefore, some researchers have designed a more reasonable Transformer network structure for the characteristics of person Re-ID tasks. Liu et al. [11] designed a trinomial Transformer model that jointly transforms person data into spatial, temporal, and spatio-temporal domains to obtain a richer and more comprehensive feature representation. To solve the problem that Transformer tends to overfit in small person datasets, Zhang et al. [12] proposed a perceptually constrained Transformer model based on loss calculation of the model in spatial and temporal dimensions. He et al. [13] used a single Transformer combined with a designed puzzle patch and an auxiliary information embedding module to form a powerful backbone network to extract discriminative features in person images and achieved better performance. Zhu et al. [14] added a learnable local Token vector to the Transformer, then they integrated local alignment into the self-attentive mechanism, so that both local features of person images are learned while the overall image matching is considered. All the above Transformer-based methods achieve high performance in person Re-ID, but the structure of these methods does not consider the hierarchical characteristics of person images, and the extracted features are incomplete. In addition, they are computationally intensive, which is not conducive to practical utilization.

Different from the above methods, this paper proposes a method based on vision Transformer with hierarchical structure and windows shifting mechanism [15] to extract person image features, which saves computational effort while expanding the perceptual field layer by layer to consider hierarchical features. Furthermore, a way to experiment and analyze the field of person Re-ID is provided.

## Methodology

### Problem definition

Deep learning-based person Re-ID methods are usually based on an effective deep learning network, which extracts image features through the network and then uses a loss function for representation or metric learning. During the learning process, assume that the training set has *n* images of *K* persons and the image *x* is input into the network *f*, the last layer of the network outputs the ID prediction vector $y=[y_{1},y_{2},\ldots ,y_{k}]\in R^{K}$ of *x*. Therefore, the probability that the image belongs to the *k*th person ID is $p\left(k\right)=\text{exp}(y_{k})/(\sum_{i=1}^{K}\text{exp}(y_{i}))$. Thus, the loss function of the network is:

$$\begin{array}{c} L\left(f,x\right)=-\sum_{k=1}^{K}q\left(k\right)\text{log}\;p\left(k\right) \end{array}$$ (1)

if the label of image *x* is equal to the predicted ID, then *q*(*k*) = 1, otherwise it is 0.

### Vision Transformer

The vision Transformer mainly implements image feature extraction by multi-head self-attention (MSA) mechanism.

**Fig 1:**
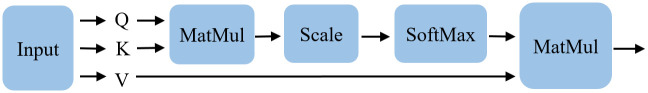
Self-attention mechanism.

**Fig 2:**
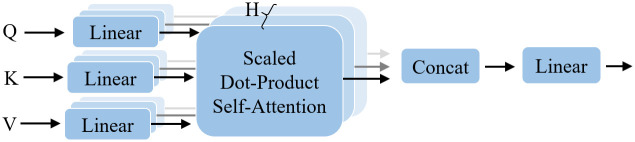
Multi-head self-attention mechanism.

According to the self-attentive operation shown in Fig 1, the input image $X\in R^{n\times d}$ is multiplied with three different weight vectors and is linearly transformed into three components, i.e., $Q\in R^{n\times d_{k}},K\in R^{n\times d_{k}}$, and $V\in R^{n\times d_{v}}$, *n* is the number of inputs *X*, *d*, *d*_*k*_, *d*_*v*_ are the dimensions of ***X***, ***Q*** and ***V***, respectively. Next, ***Q*** and ***K*** are matched as an inner product. Next, the inner product result is scaled and fed into the Softmax function for normalization. If the input of Softmax is not scaled, the gradient of Softmax will tend to zero in case the input has a large order of magnitude, causing the gradient to vanish. Then, the output of Softmax is the self-attentive output of ***Q***, and this output is accumulated as the weights of ***V***. Finally, the output of the self-attentive matrix is obtained and defined as

$$\begin{array}{c} \text{Attention}\left(Q,K,V\right)=\text{Softmax}(\frac{QK^{T}}{\sqrt{d_{k}}})V \end{array}$$ (2)

where $\sqrt{d_{k}}$ is a scaled factor that enhances the normalization operation. MSA splits ***Q***, ***K*** and ***V*** into *H* heads as presented in Fig 2, the self-attention operations are performed in parallel, and then the output of each head is concatenated to form the final output. The headers are defined as

$$\begin{array}{c} \;\text{head}{\;}_{i}=\text{Attention}(QW_{i}^{Q},KW_{i}^{K},VW_{i}^{V}) \end{array}$$ (3)

where $W_{i}^{Q}\in R^{dxd_{k}}$, $W_{i}^{K}\in R^{dxd_{k}}$, $W_{i}^{V}\in R^{dxd_{k}}$, *i* ∈ [1, *H*].

The output of the MSA operation is

$$\begin{array}{c} \;\text{MultiHead}\;\left(Q,K,V\right)=\text{Concat}({\text{head}}_{1},\ldots ,\;\text{head}{\;}_{H})W^{0} \end{array}$$ (4)

where $W^{0}\in R^{hd_{v}\times d}$ is the parameter matrix and *H* is the number of heads.

In this paper, the mechanism of preserved MSA allows the matrix representing the same image to form multiple subspaces with the same size of the overall matrix. Only the size of the dimension corresponding to each attention head is changed, which allows the image matrix to learn information on multiple aspects while the computational effort is consistent with that of a single self-attention head.

### Proposed method

**Fig 3:**
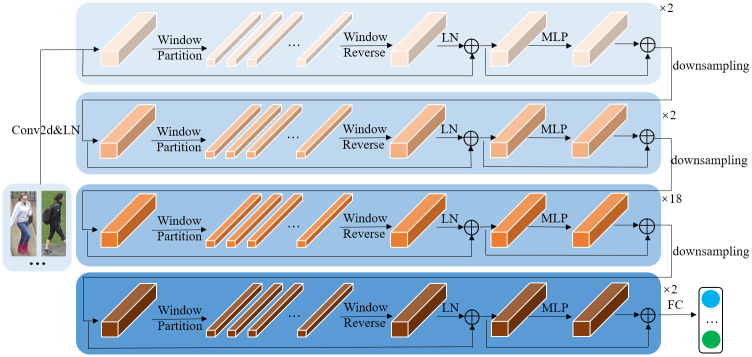
Overall model architecture.

In general, integrating hierarchical multiscale features can improve the performance of models in the field of image classification. However, the person Re-ID task is more special, it requires a large number of features with discriminative properties. The traditional low-level and high-level feature aggregation approaches could limit the performance of the model with less feature information, so the proposed method aims to combine the hierarchical features from a global perspective, and the network architecture used in this paper is shown in Fig 3.

Unlike the general downsampling approach, this paper divides the image into different layers according to different size of 4 × 4, 8 × 8, 16 × 16, and 32 × 32 patches, so as to achieve a hierarchical arrangement of feature extraction and thus achieve an overall hierarchical distribution of features. For a person image with the size of H × W × 3, the image is first cut into 4 × 4 patch and then embedded into a *C*-dimensional vector by convolution, so that the feature dimension of each patch is 4 × 4 × 3 = 48. After that, a regular window is set by window partition, i.e., the window is divided evenly. Then, a vision Transformer is used inside the window, and the information between patches can be obtained by MSA operation.

**Fig 4:**
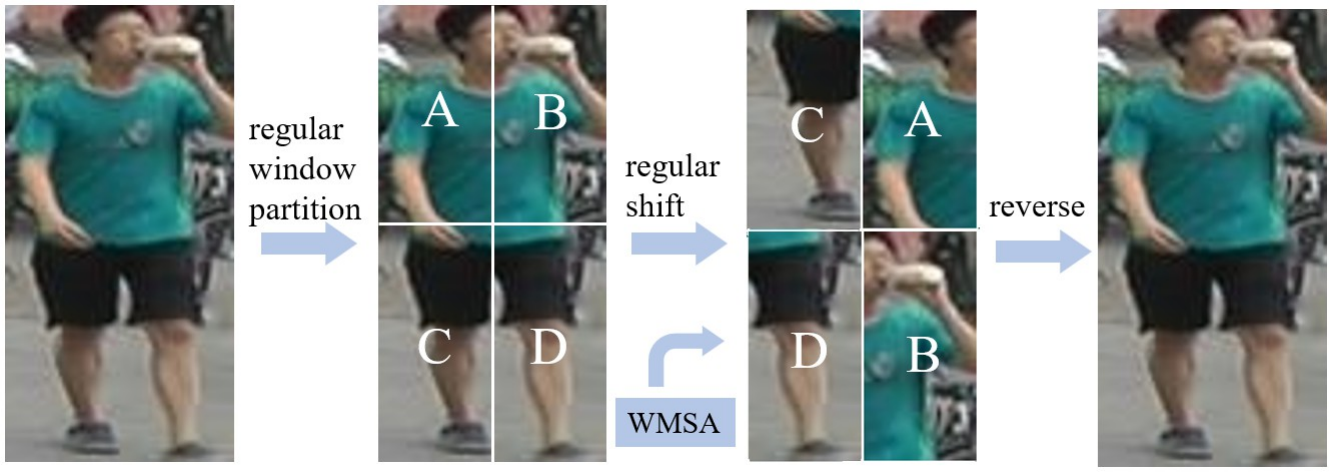
The process of regular window partition and reverse.

As shown in Fig 4, to let different vectors learn richer attention information, this paper performs a regular shift of the divided windows and then does another MSA operation. Next, in order to be able to get the complete image information, this paper aggregates the divided windows into a complete vector by reversing the cycle. Then, the feature vectors during the training process by layer normalization (LN) and multilayer perceptron (MLP) optimization are updated. LN plays a key role in stabilizing model training and maintaining model convergence, for a given image $x\in R^{d}$,

$$\begin{array}{c} LN\left(x\right)=\frac{x-\mu }{\delta }\circ \gamma +\beta \end{array}$$ (5)

where μ∈R and δ∈R are the mean and standard deviation of the features, respectively. ∘ is the dot product operation, $\gamma \in R^{d}$ and $\beta \in R^{d}$ are the learnable model parameters. MLP is used for feature transformation and nonlinear mapping and is defined as

$$\begin{array}{c} MLP\left(X\right)=\sigma (XW_{1}+b_{1})W_{2}+b_{2} \end{array}$$ (6)

where $W_{1}\in R^{d\times d_{m}}$ and $W_{2}\in R^{d_{m}\times d}$ are the weight matrices of the two fully connected layers, $b_{1}\in R^{d_{m}}$ and $b_{2}\in R^{d_{m}}$ are the bias terms, and *σ*(•) is the GELU activation function.

**Fig 5:**
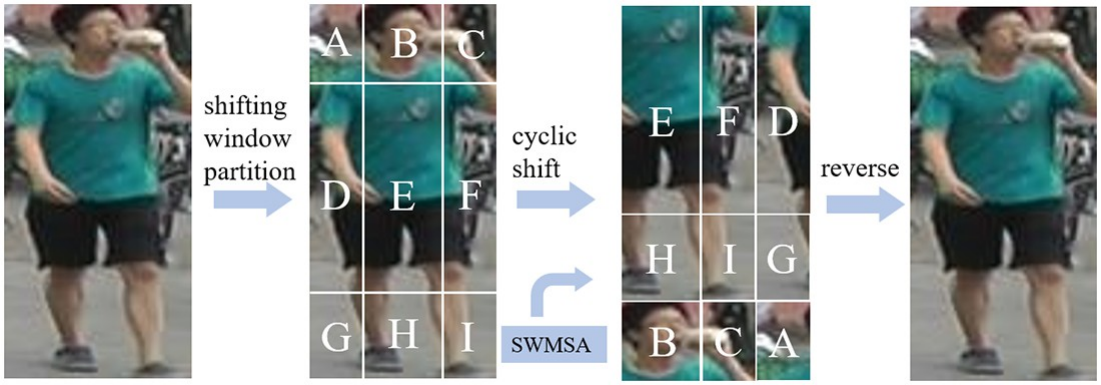
The process of shifting window partition and reverse.

After the regular window is delineated, the shifting window is used to optimize the feature vector again. The regular window is divided into 4 chunks of 2 × 2 size, each size of which is *M* × *M*. Yet, the shifting window is divided into 3 × 3 windows of different sizes by keeping the middle part of the image *M* × *M* size unchanged and dividing the windows at the edges of the image with an even ratio of minimum *M*/2 and maximum *M* size, which makes the adjacent non-overlapping regular windows in the upper layer connected to each other and increases the perceptual field. The process of shifting window partition and reverse is shown in Fig 5.

The self-attention operation within the regular window is denoted as WMSA (Windows MSA) and the self-attention operation within the shifting window is denoted as SWMSA (Shifted Windows MSA), then the operations of these two layers are:

$$\begin{array}{c} {\hat{z}}^{l}=WMSA(LN(z^{l-1}))+z^{l-1} \end{array}$$ (7)

$$\begin{array}{c} z^{l}=MLP(LN(z^{l}))+{\hat{z}}^{l} \end{array}$$ (8)

$$\begin{array}{c} {\hat{z}}^{l+1}=SWMSA(LN(z^{l}))+z^{l} \end{array}$$ (9)

$$\begin{array}{c} z^{l+1}=MLP(LN({\hat{z}}^{l+1}))+{\hat{z}}^{l+1} \end{array}$$ (10)

where ${\hat{z}}^{l}$ and *z*^*l*^ are the outputs of WMSA and MLP for the regular window, respectively; ${\hat{z}}^{l+1}$ and *z*^*l*+1^ are the outputs of SWMSA and MLP for the shifting window, respectively.

In general, the person images are firstly pre-processed, then the model is trained on the training set images, and finally the performance of the model is evaluated in the test set. In this paper, the whole model adopts a hierarchical design, which consists of four stages of layering and window shifting Transformer encoding. In addition to the first stage of encoding, each stage expands the perceptual field layer by layer by downsampling in order to obtain the global information. The overall training pseudo-code of our model is shown in the following.

**Algorithm 1** Vision Transformer with Hierarchical Structure and Windows Shifting for Person Re-identification

**Input:** Training dataset *S*, training epoch *p*

**Output:** Upadated model *f*, predicted label vector *y*

1: The order of *S* is randomly disordered and pre-processed with data enhancement, normalization, etc.

2: **for** epoch = 0: *E*-1 **do**

3:  **for** *L* = 0: 3 **do**

4:   *S* is split according to the patch size of 4 × 2^*L*^ × 4 × 2^*L*^

5:   The split patch is windowed and the Transformer output is calculated according to Eqs 2 ∼ 4

6:   The output of Transformer is optimized using Eqs 5 and 6

7:   Window shifting and the output of Transformer are calculated according to Eqs 2 ∼ 4

8:   The output of Transformer optimized using Eqs 5 and 6

9:  **end for**

10: The Transformer output after 4-layer optimization is predicted according to Eq 1

11: **end for**

## Experiments

### Datasets and evaluation metrics

In this paper, three publicly available datasets commonly used for person Re-ID are selected for experimental validation, which are Market-1501 [16], DukeMTMC-reID [17] and MSMT17 [18].

The Market-1501 dataset contains person images that was collected by a total of 6 cameras in Tsinghua University campus. These images contain 32,668 persons with 1501 IDs. Among them, 751 persons were assigned to the training set with a total of 12,936 images, with an average of 17.2 training images per person. There were 750 persons in the test set containing 19,732 images, with an average of 26.3 test images per person. One image was randomly selected as a query in each camera, so there were up to 6 queries for one person, and the query set totaled 3,368 images.

DukeMTMC-reID was collected at Duke University with images from 8 different cameras. The training set has 16,522 images containing 702 persons, with an average of 23.5 training images per person. The test set of 702 persons contains 17,661 images, with an average of 25.1 test images per person. The 702 people in the test set randomly selected one image from each camera as a query, with a total of 2,228 images.

The MSMT17 dataset captured 126,441 person images from 15 cameras, with a total of 4,101 different persons. Among them, the training set contains 1,041 persons with a total of 32,621 images, and there is an average of 31.3 training images per person; the test set contains 3,060 persons that make up a total of 93,820 images, with an average of 30.6 test images per person.

In the evaluation of experimental results, this paper uses the Rank-k metric from the Cumulative Matching Characteristics (CMC), which uses the highest scoring label as the predicted label to calculate accuracy. In practical use, the more representative Rank-1 value is usually chosen to replace the CMC curve. In addition, Mean Average Precision (mAP) is another important evaluation metric that can more robustly reflect the performance of the model. The mAP metric has an upper limit of 1 and a lower limit of 0. The stronger the person Re-ID model is, the higher the mAP value is.

### Parameter setting

For data preprocessing, all person image size is uniformly adjusted to 224 × 224, and then a value of 0 is filled with 10 pixels at the edges of the rescaled images. Next, these images are randomly cropped into a rectangular box of 224 × 224 and flipped horizontally with a probability of 0.5. Finally, each person image is decoded with 32-bit floating point in the range of [0, 1], and the RGB channels are normalized by subtracting 0.485, 0.456, 0.406 and dividing by 0.229, 0.224 and 0.225, respectively. For the model training parameter, the model parameters are updated with SGD optimizer and learning rate of 0.01 in this paper. In addition, the batch size is 32 and the total epoch is 60. For the experimental environment, the pytorch framework and one NVIDIA RTX 2060 GPU are used for model training.

### Parameter setting

#### Comparison with different baselines

In order to evaluate the performance of the methods in this paper more intuitively and comprehensively, state-of-the-art implementations containing CNN-based, GAN-based, CNN+Attention-based, and Transformer-based baselines are selected. The mAP (%) values and Rank-1 (%) values of the compared methods based on three datasets, Market-1501 and DukeMTMC-reID, are shown in Table 1. The comparison methods include CNN-based MGN [19], Pyramid [20], SNR [21]; GAN-based mGD+RNLSTM [22], JoT-GAN [23]; CNN+Attention-based IAPM [24], SONA [25], ABD-Net [7], RGA-SC [26], APNet [27] and Transformer-based PAT [28], AAformer [14], NFormer [29].

**Table 1:** Performance comparison of our method with baselines on the Market1501, DukeMTMC-reID and MSMT17 dataset.

Types	Methods	Market1501		DukeMTMC-reID		MSMT17	
		mAP	Rank-1	mAP	Rank-1	mAP	Rank-1
CNN	MGN	86.90	95.70	78.40	88.70	-	-
	Pyramid	88.20	95.70	79.00	89.00	-	-
	SNR	84.70	94.40	73.00	85.90	-	-
GAN	mGD+RNLSTM	77.90	91.30	63.90	80.80	-	-
	JoT-GAN	87.60	95.10	77.00	88.00	50.14	73.71
CNN+Attention	IAPM	86.30	95.20	75.70	88.00	-	-
	SONA	88.80	95.60	78.30	89.40	-	-
	ABD-Net	88.30	95.60	78.60	89.00	60.80	82.30
	RGA-SC	88.40	96.10	-	-	57.50	80.30
	APNet	88.40	96.10	-	-	59.00	80.80
Transformer	PAT	88.00	95.40	78.20	88.80	-	-
	AAformer	87.70	95.40	80.00	90.10	63.20	83.60
	NFormer	91.10	94.70	83.50	89.40	59.80	77.30
	Ours	89.30	95.40	81.20	90.40	63.84	83.79

From Table 1, it can be seen as follows:

1. The optimal method based on CNN is the Pyramid. The mAP values on the Market1501 and DukeMTMC-reID are 88.20% and 79.00%, respectively, which are 1.1% and 2.2% different from the performance of our method. It shows that the attention mechanism is added to the hierarchical feature extraction of our method has played a role in promoting person Re-ID. Meanwhile, the network considers the relationship between person image information and improves the generalization ability.
2. The mAP values of the optimal method based on GAN are 1.7% and 4.2% in the Market1501 and DukeMTMC-reID less than our method, respectively, indicating that our method has enough information to extract features, and has higher performance without additional generated image sets.
3. In the method based on CNN + Attention, APNet has the best mAP values on the Market1501 and DukeMTMC-reID datasets with 89.00% and 78.80%, respectively, but is inferior to ABD-Net on the MSMT17 dataset. The mAP values of APNet differes from our method by -0.3% and 1.4% on the first two datasets, respectively. And ABD-Net differes from the mAP values of our method by 3.04% on the MSMT17 dataset. It indicates that in the case of the same hierarchy and mutual information, the hierarchy and window shifting mechanism of our method can further obtain the information within the person image, and finally the person retrieval results are improved effectively.
4. In the Transformer-based methods, the mAP values of NFormer on the Market1501 and DukeMTMC-reID datasets are 91.10% and 83.50%, respectively, which are -0.8% and -2.3% different from the performance of our method. However, the mAP values on the MSMT17 dataset differ from our method by 4.04%, and the suboptimal AAformer is also lower in performance than our method. It shows that the hierarchy and window shifting mechanism used in this paper complements the global features on the basis of Transformer, and finally more discriminative features are formed. In summary, our method can effectively aggregate shallow detail information and deep depth information to perform person Re-ID tasks.

#### Process analysis

**Fig 6:**
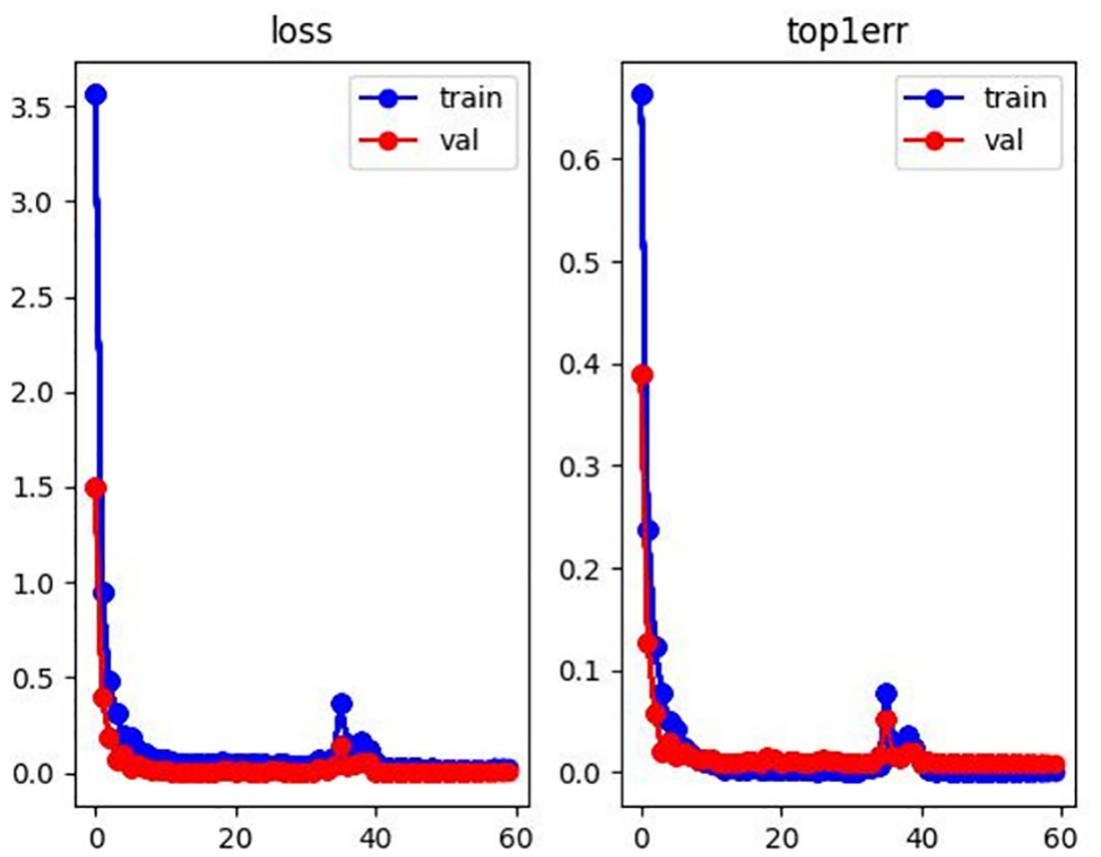
Loss and top1 error curve with Market1501 dataset.

**Fig 7:**
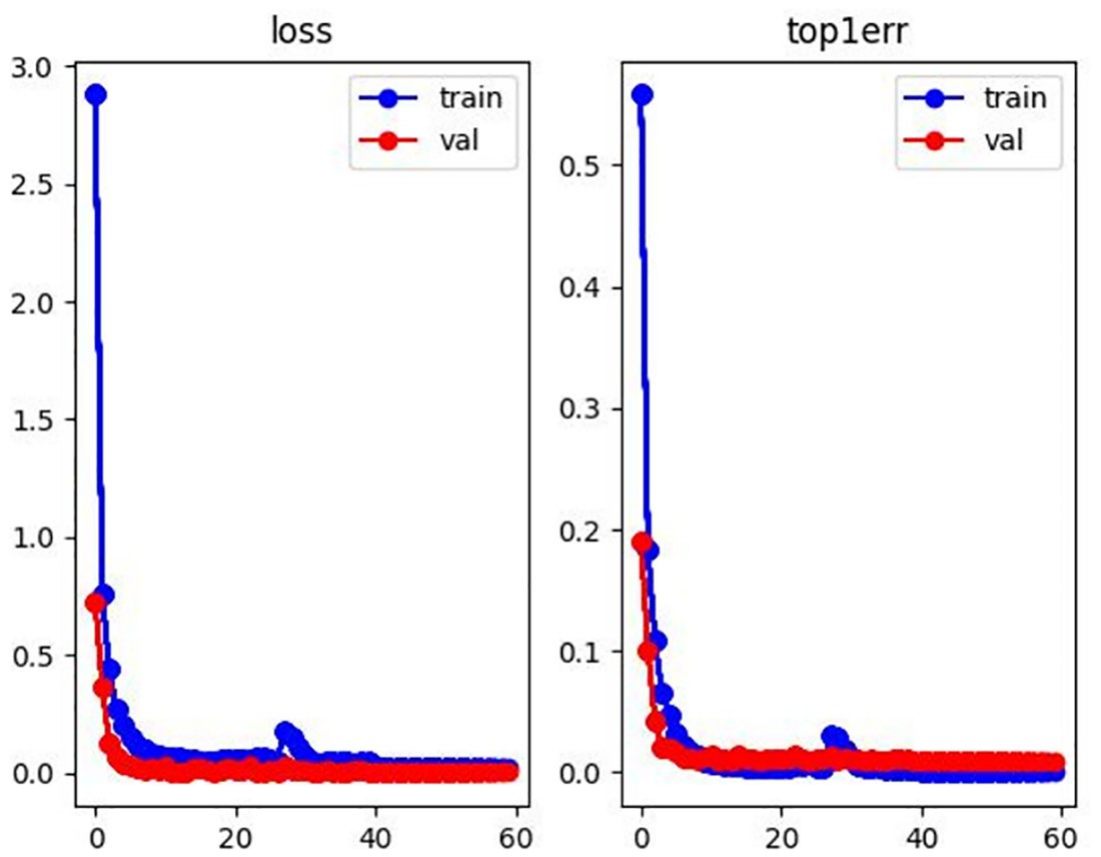
Loss and top1 error curve with DukeMTMC-reID dataset.

**Fig 8:**
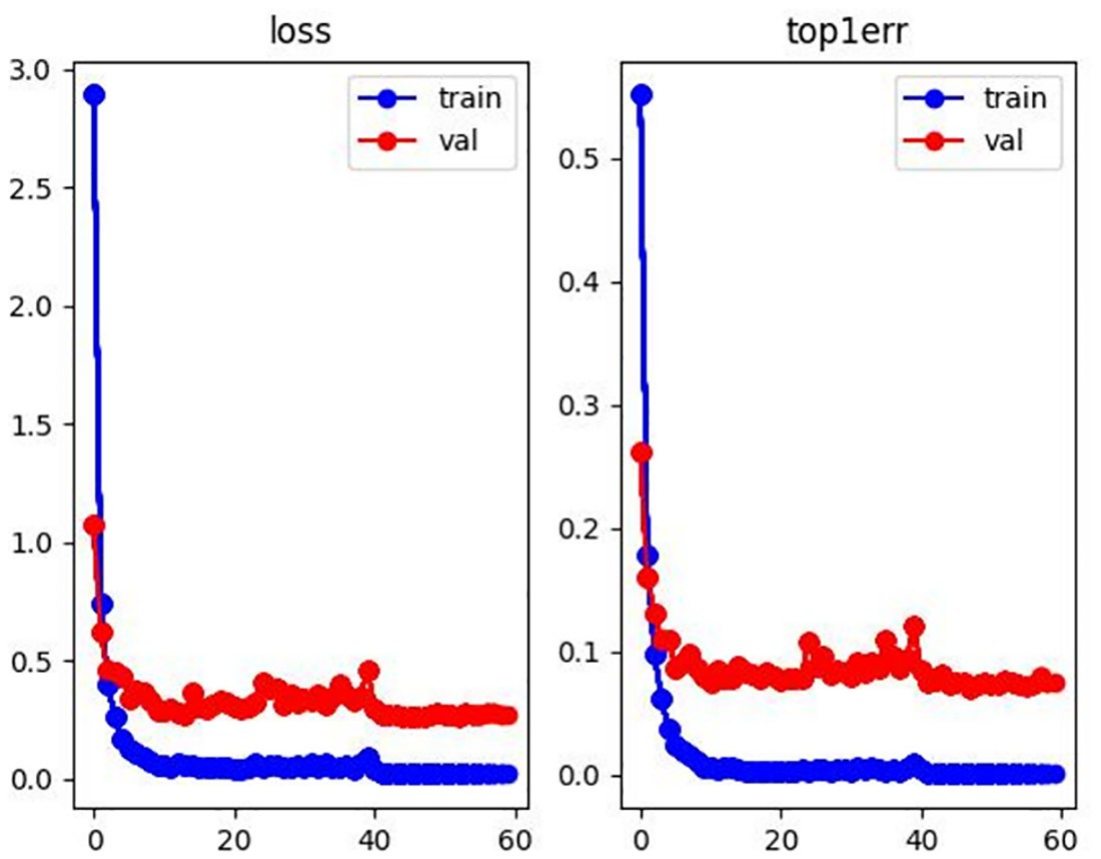
Loss and top1 error curve with MSMT17 dataset.

In order to further illustrate the accuracy of the results, this paper visualizes the loss curve and top1 errors in the training processing as shown in Figs 6–8. The horizontal axis represents epoch, and the vertical axis represents the corresponding value. It can be seen from Figs 6–8 that the model achieves optimal and stable performance in predicting the identity of each person after 60 epochs of training.

**Fig 9:**
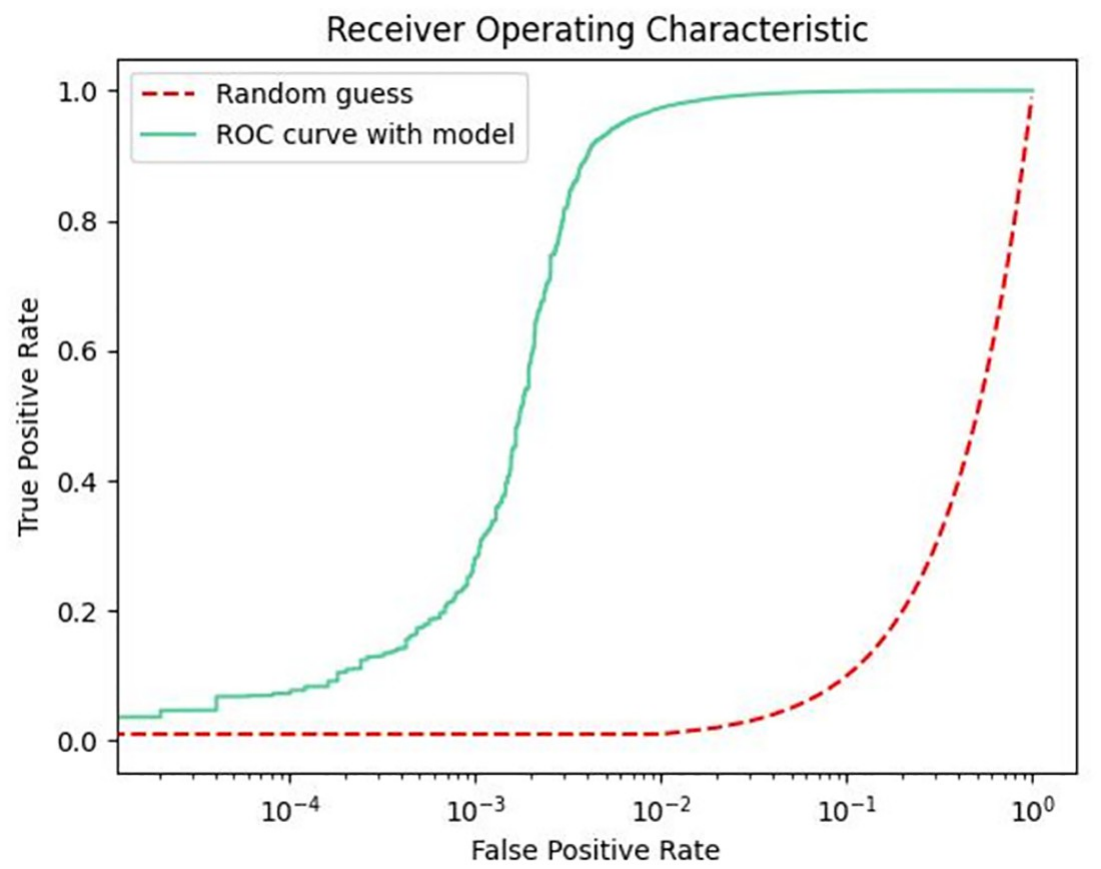
ROC curve with Market1501 dataset.

**Fig 10:**
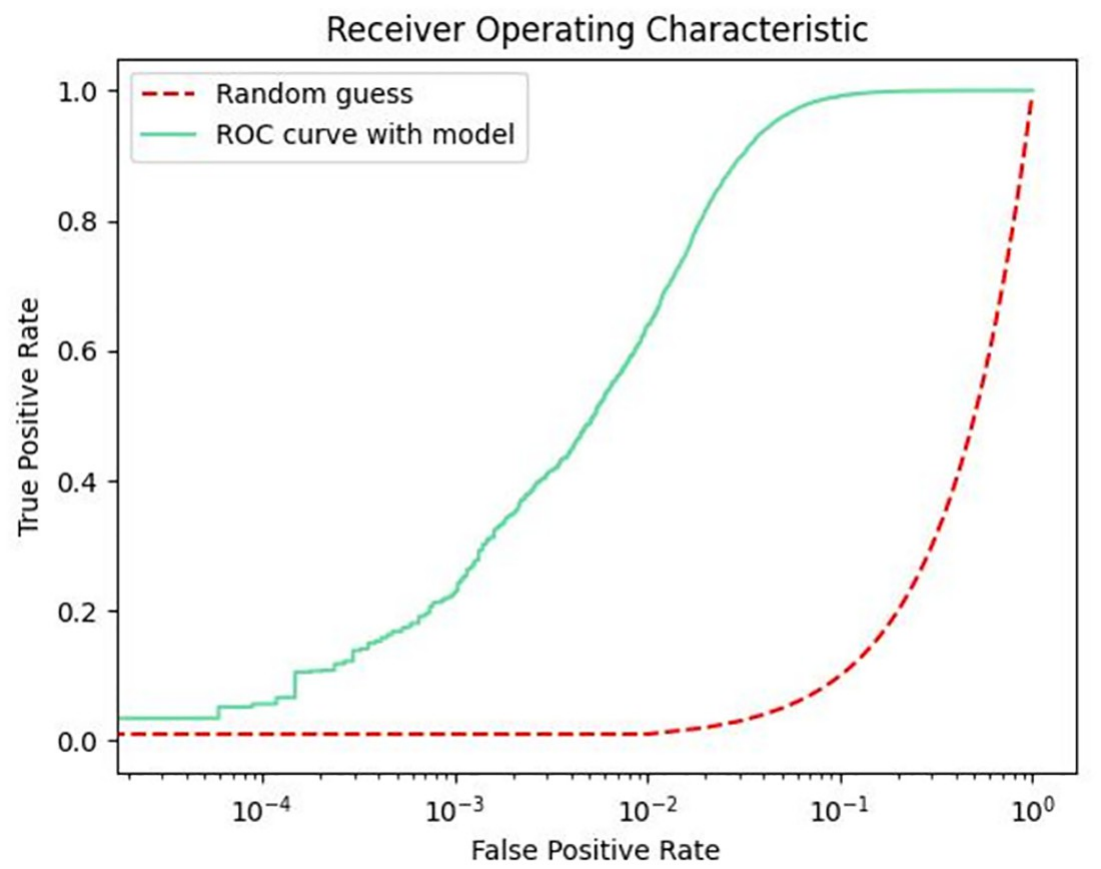
ROC curve with DukeMTMC-reID dataset.

**Fig 11:**
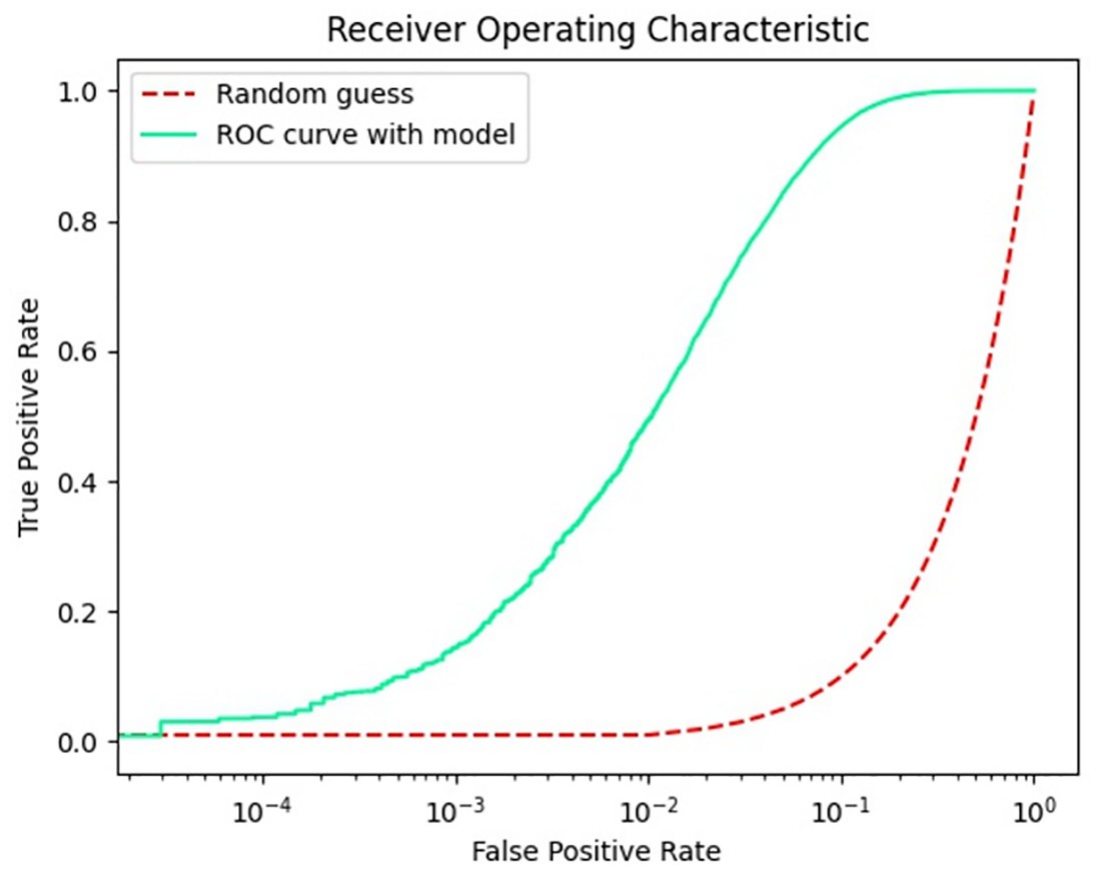
ROC curve with MSMT17 dataset.

The ROC curve can be used to evaluate the credibility of the model classifier, so this paper presents the performance of the trained model in the test set in the form of ROC curve. As shown in Figs 9–11, in the non-uniform interval between 0 and 0.1, the correct rate of model prediction is still increasing. In the interval between 0.1 and 1, the correct rate of model prediction tends to be stable. This indicates that our model performs stably in predicting performance for person ID.

**Fig 12:**
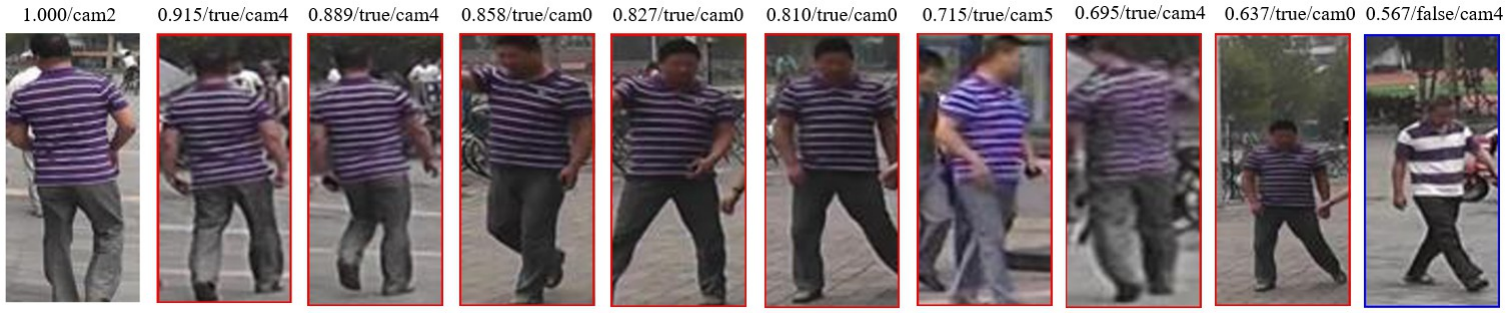
Example 1 of ranking results.

**Fig 13:**
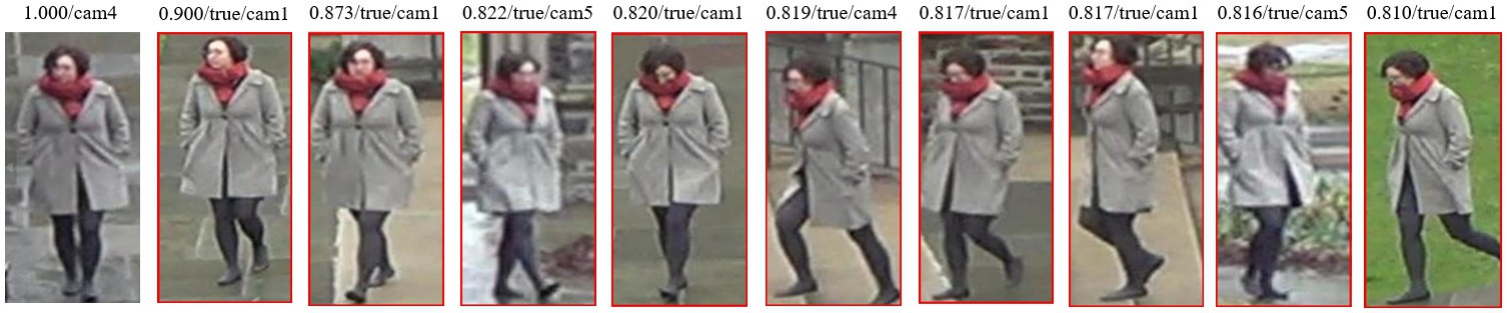
Example 2 of ranking results.

**Fig 14:**
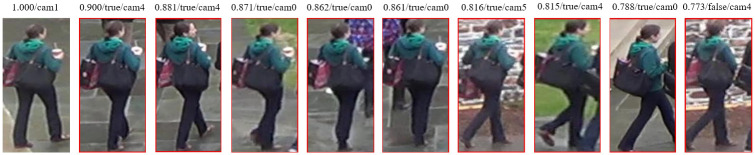
Example 3 of ranking results.

After presenting the overall performance improvement of the model, this paper also shows three rank list examples of the model on the test set, as shown in Figs 12–14. The first column is the original person image, and the columns 2 to 9 are the person images found in other cameras that are most similar to the original person image. Ranked by cosine similarity with the original person image, the similarity values are labeled on each person image. This paper shows an incorrect prediction in the first person ranking example, which shows that the model cannot predict all person identities completely correctly and occasionally mispredicts them.

**Fig 15:**
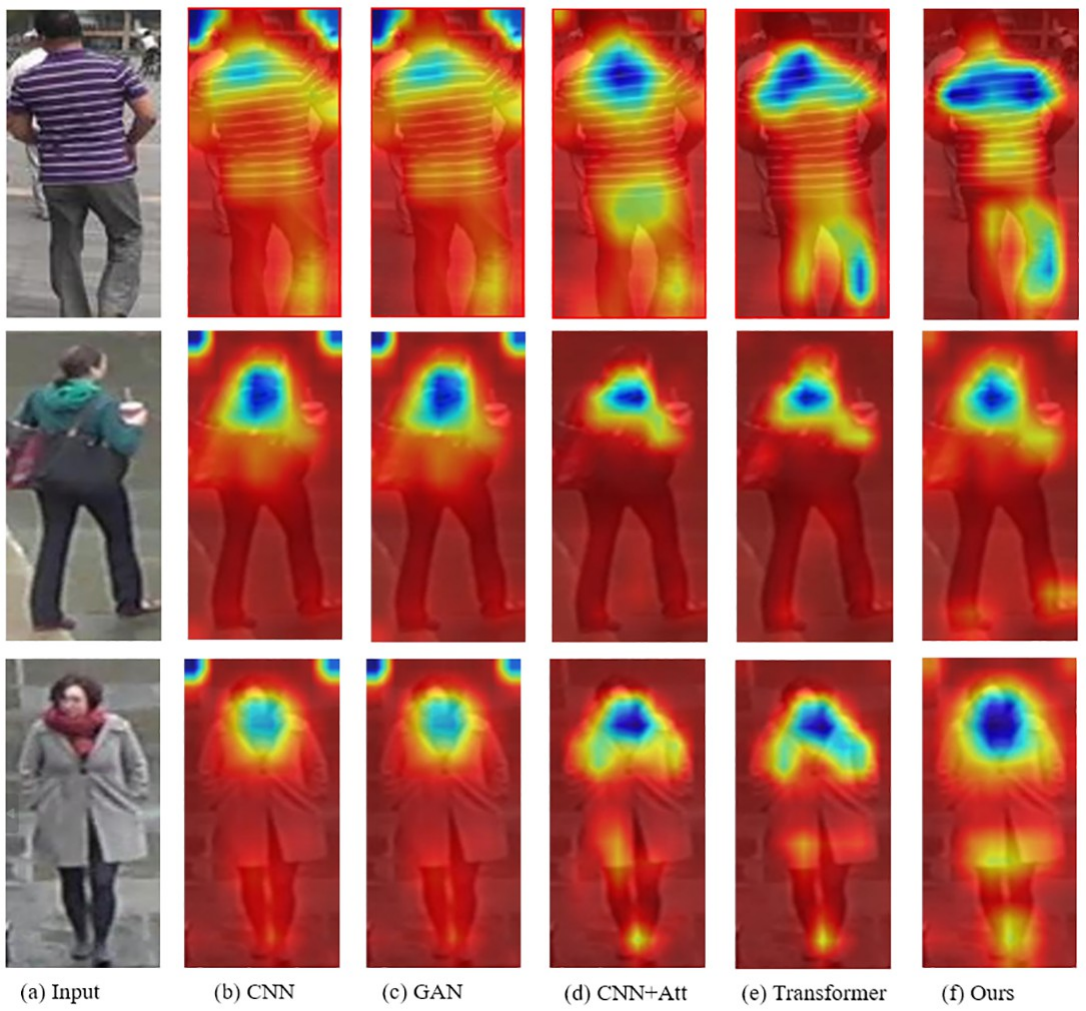
The examples of the feature visualization for different methods.

In addition to demonstrating the model performance from the perspective of similarity ranking visualization, this paper also compares the visualization features of different models to more intuitively illustrate the superiority of the method in this paper, as shown in Fig 15. Among them, both the CNN-based method and the GAN-based method use the backbone of CNN, so they have the same visualization results.

#### Ablation study

In order to verify the effectiveness of the hierarchy and window shifting, this paper first conducts experiments on the regular window partition and uses it as a baseline, and then experiments on the baseline + hierarchy, baseline + window shifting, baseline + hierarchy + window shifting, respectively. The results are shown in Table 2.

**Table 2:** Ablation experiments of our method on the Market1501, DukeMTMC-reID and MSMT17 datasets.

Types	Market1501		DukeMTMC-reID		MSMT17	
	mAP	Rank-1	mAP	Rank-1	mAP	Rank-1
baseline	87.85	93.22	79.31	88.67	60.45	80.17
baseline + hierarchy	88.12	94.57	80.18	89.35	62.30	81.72
baseline + window shifting	88.36	94.74	80.33	89.74	63.57	82.03
baseline + hierarchy + window shifting	89.30	95.40	81.20	90.40	63.84	83.79

From Table 2, it can be seen that the performance of the baseline method without hierarchy and window shifting is similar to that of the traditional CNN-based method, and adding hierarchy or window shifting to the baseline has a significant performance improvement, while the mAP values of adding hierarchy and window shifting exceed those of the baseline method by 1.45% and 1.89%, respectively. The experiments show that the approach with hierarchy and window shifting outperforms the general Transformer model in terms of overall feature representation of person images.

#### Complexity analysis

In order to verify the efficiency of the method used in this paper, the analysis is performed from the basis of CNN, GAN, CNN+Attention, and Transformer networks. Assuming that both input and output size are *n* × *d*, in the case of convolutional kernel size is *k* for CNN, in order to ensure that the input and output are the same in the first dimension, there is usually fill operation, so the actual convolutional kernel size is *k* × *d*. At this time, the complexity of one operation is O(kd), and a total of *n* times operations are done, so the complexity is O(nkd). In order to ensure the uniformity of the second dimension, *d* convolution kernels are needed, so the total time complexity of the convolution operation is $O(nkd^{2})$. Similarly, the GAN-based feature extraction cited in this paper is based on a CNN, so the time complexity is the same as that of the CNN. The total time complexity of CNN+Attention is $O(nkd^{2}+n^{2}d)$, which is due to the fact that the regular attention mechanism can be viewed as the multiplication of two matrices of size (*n*, *d*) and (*d*, *n*) when computed. Therefore, the time complexity of the attention mechanism is $\left(n,d\right)*\left(d,n\right)=O\left(n^{2}d\right)$. Adding the complexity of CNN, the total time complexity is $O(nkd^{2}+n^{2}d)$. Transformer performs MSA for all patches, so the total time complexity is $O(n^{2}d+nd^{2})$. Our method splits *n*/*m* (*m* is a constant) patches into multiple groups, WMSA is performed between patches within the group, so the total time complexity is $O(n^{2}d+nd)$. Compared with the traditional Transformer model, our method has a smaller time complexity, and this grouping calculation method can also reduce the amount of calculation.

In addition to the complexity analysis of several methods in theory, the experimental results about the number of floating-point operations (FLOPs) and the time used to complete one recognition for each person image are given in Table 3. Among them, CNN and GAN are based on the ResNet50 architecture, and all models are experimented using Eq 1 as the loss function.

**Table 3:** Comparison of computation efficiency among different methods.

Methods	FLOPs	Time
CNN	3.84 × 10^9^	1.2s
GAN	3.84 × 10^9^	1.4s
CNN + Attention	4.63 × 10^9^	2.8s
Transformer	8.91 × 10^9^	17.5s
Ours	5.77 × 10^9^	14.7s

From Table 3, our method is smaller in terms of FLOPs than Transformer and running time, while consistent with the theoretical analysis is that the time complexity is higher than the other three methods with simpler network structures.

## Conclusion

Aiming at the problem that traditional CNN-based methods ignore local area information leads to incomplete feature extraction when processing person images, we propose a person Re-ID method based on vision Transformer by introducing hierarchical structure and window shifting, which enhances the ability to extract complete features of person images. Theoretical derivation and experimental analysis show that our method is able to learn information across windows by delineating windows. In addition, the downsampling enables the model to acquire multi-hierarchy person image features, and the integrity of feature extraction is better expressed by focusing on global information while considering local information. Furthermore, the proposed method provides an experimental and analytical reference for different domain practice processes. The perceptual field calculation based on the Transformer method is dynamically transformed based on the content, so there is much more space available for representation than CNN with finite weights, which leads to the method’s reliance on a large amount of data to achieve superior performance. Future research can focus on how to reduce the Transformer model’s dependence on data while maintaining excellent model performance.
